# Buccolingual Inclination Control of Upper Central Incisors of Aligners: A Comparison with Conventional and Self-Ligating Brackets

**DOI:** 10.1155/2018/9341821

**Published:** 2018-11-29

**Authors:** Maria Francesca Sfondrini, Paola Gandini, Tommaso Castroflorio, Francesco Garino, Luca Mergati, Krizia D'Anca, Federico Trovati, Andrea Scribante

**Affiliations:** ^1^Unit of Orthodontics and Paediatric Dentistry, Section of Dentistry, Department of Clinical, Surgical, Diagnostic and Paediatric Sciences, University of Pavia, Italy; ^2^Orthodontics Unit, Dental School, Department of Surgical Sciences, University of Torino, Italy; ^3^Private Practice, Torino, Italy

## Abstract

**Objective:**

The upper incisors torque expression is essential for the orthodontic treatment accuracy. Various orthodontic devices are claimed to have different inclination control capacity. The purpose of this retrospective study was to compare the radiographic buccolingual inclination of upper incisors in patients treated with three different orthodontic techniques.

**Material and Methods:**

Conventional brackets (Victory, 3M), self-ligating appliances (Damon Q, Ormco), and aligners (Invisalign, Align Technology) were tested. Cephalometric data of 25 patients with similar skeletal and dental pretreatment parameters were collected for each technique. Position changes of upper central incisors were assessed with radiographic evaluation before and after therapy. Three different parameters were considered: 11^∧^SnaSnp, 11^∧^Ocl and I+ TVL. All variables were measured before (T0) and after (T1) treatment and their variation over treatment was assessed.

**Results:**

When evaluating angular measurements, 11^∧^SnaSnp and 11^∧^Ocl angles showed the highest numeric variation with conventional brackets. Lowest values were reported with aligners. However, the differences among various techniques were not significant for both angles (P>0.05). Also I+ TVL linear value variation did not show significant differences among the different groups tested (P>0.05).

**Conclusion:**

Conventional multibrackets appliance showed the highest incisal position variations over treatment, but the differences among various groups were not significantly different.

## 1. Introduction

The expression of torque is one of the most important key factors in orthodontic treatment. Clinically, the torque represents the third key of occlusion, described as the buccolingual inclination of the dental crown [1]. The optimal torque is related to a correct anterior guidance, proper distribution of the arch spaces, and appropriate overjet and overbite [2]. Moreover, in the anterior sector of upper arch, an adequate torque value has a significant impact on smile aesthetics and soft tissues profile. A limited torque control in the anterior area causes shortening of the dental arch, possible occlusal interferences, and a narrower smile [3].

The conventional multibrackets technique is the most widely studied orthodontic appliance and it has been described as a tool with efficient torque control capability [4, 5]. Since the torque expression mainly depends on the bracket/archwire interaction, several factors, such as the amount of torsion, the size and quality of the wire, the play of the wire in the bracket slot, the angulation, and the deformability of the bracket could produce important variations [6, 7].

In order to reduce chairside time and bracket/wire friction, self-ligating brackets have been introduced in orthodontic practice [8–10]. These appliances allow brackets to engage the wire by means of a sliding mechanism [11]. They present advantageous characteristics, such as efficient orthodontic mechanics, lower friction, less chair time, and good patient satisfaction [12–14]. However, in an in vitro study, self-ligating brackets seem to be related to a higher torque loss if compared with conventional brackets [1].

The growing aesthetic demands of orthodontic patients led the research towards the development of more aesthetics and comfortable orthodontic appliances [15]. In the late nineties thermoplastic aligners, based on computer aided design procedures have been introduced [16, 17].

Despite claims about the capability of aligners, some authors state that evidence is generally lacking. Shortened treatment duration and chair time in mild-to-moderate cases appear to be the only significant effectiveness of aligners [17]. Even if the consumer demand of these devices is constantly increasing, their efficacy and accuracy in torque control remain somewhat unexplored [18].

Therefore, the aim of this retrospective clinical study was to evaluate the effectiveness of conventional brackets, self-ligating bracket, and aligners on torque expression, evaluating lateral cephalometric values in an orthodontic population. The null hypothesis of the study was the absence of significant difference in incisal inclination variation during therapy among the three different techniques.

## 2. Materials and Methods

Three different orthodontic techniques were evaluated: conventional brackets (Victory; 3M, Monrovia, California, USA), self-ligating appliances (Damon Q, Ormco, Orange, California, USA), and aligners (Invisalign, Align Technology, Santa Clara, California, USA).

The present study followed Helsinki Declaration. Internal Unit Review Board accepted the study design (Ref: 16-0318).

25 patients for each technique were retrospectively selected (overall mean age 25.5 ± 6.5 years, Mean Index of Treatment Needs: 3).

Inclusion criteria involved permanent teeth, dental class I, or mild classes II and III and need for upper incisal torque change. Exclusion criteria were the presence of anterior or posterior cross bites, skeletal class III, severe skeletal deformity, previous orthodontic therapies, skull/facial traumas, and temporomandibular disorders.

Maximum torque expression was obtained by a 0.019x0.025-inch stainless steel archwires (3M, Monrovia, USA) for conventional and self-ligating brackets, whereas for aligners by the Power Ridge technology (Power Ridges, Align Technology, Santa Clara, California, USA).

For each patient, lateral cephalometric radiographs before (T0) and after therapy (T1) were collected and computerized cephalometric traces were performed by the same operator twice, with an interval of two weeks (software Deltadent 1.0, Milan, Italy).

An expert operator in the technique treated every group of patients.

The following points were considered for each patient's radiographs: upper incisal point (INI+), upper apical point (API+), anterior nasal spine (Sna), posterior nasal spine (Snp), occlusal anterior point (Ocla), occlusal posterior point (Oclp), gonion (Go), menton (Me), orbitale (Or), porion (Po), sella (S), A point (A), and B point (B). For each X-ray, the following plans were traced: upper incisal axis (INI + - API +); palatal plane (Sna - Snp); occlusal plan (Ocla - Oclp); mandible plane (Go - Me); Frankfurt horizontal plane (Or - Po); TVL (True Vertical Line); S-N; N-A; and N-B planes. The same operator performed all the tracings.

The following angles were employed in order to evaluate the homogeneity of the patients enrolled and to eliminate possible sample bias: ANB and Wits index (to assess the skeletal class); SnaSnp^∧^GoMe (to identify the divergence).

The torque evaluation was performed testing the variation over treatment of the following variables: 11-Sna^∧^Snp (angle formed by the upper incisal axis with the palatal plane); 11^∧^Ocl (angle determined by the axis of the upper incisor and the occlusal plane); and I + TVL (linear distance of the most advanced point of the vestibular surface of the upper incisor from the TVL).

Data analysis was performed with software (R version 3.1.3, R Development Core Team, R Foundation for Statistical Computing, Wien, Austria). To estimate the method error, the same operator retraced radiographs after a period of 6 weeks, measures were assessed with t-test, and no significant variations were reported between two groups. Descriptive statistic of the different variables (mean, standard deviation, median, minimum and maximum values) was computed for each group. The normality of distributions was evaluated by Kolmogorov and Smirnov tests. Subsequently an ANOVA test was applied followed by the Tukey test as a post hoc test. For all tests the significance was set for P <0.05.

## 3. Results

No significant differences (P>0.05) were observed among the three different groups when evaluating pretreatment skeletal and dental parameters (Table 1).

The variation of the upper incisal inclination and, consequently, the expression capacity of the upper incisors torque was evaluated at T0 and T1 using three different values: $11\;\hat{\;}\;SnaSnp$, $11\;\hat{\;}\;Ocl$ and I + - TVL (Table 2). The variation over treatment of the three variables was compared.

**Figure 1:**
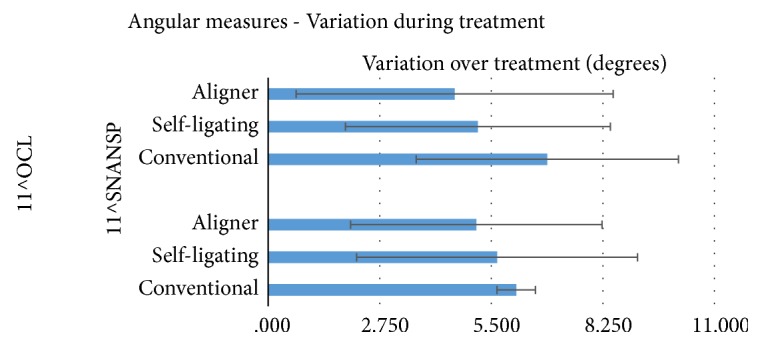
Variation over treatment of upper incisor angular measures (11^∧^OCL and 11^∧^SNASNP) using the three different orthodontic techniques.

**Figure 2:**
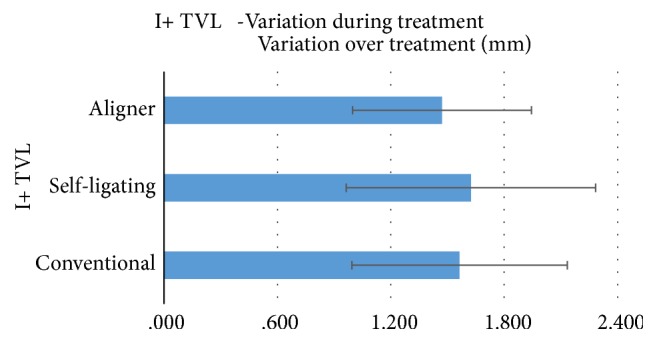
Variation over treatment of upper incisor linear measure (I+TVL) using the three different orthodontic techniques.

When evaluating angular measurements, 11^∧^SnaSnp and 11^∧^Ocl angles showed the highest numeric variation with conventional brackets. Lowest values were reported with aligners (Figure 1). However, the differences among various techniques were not significant for both angles (P>0.05). In addition, I+ TVL linear value variation (Figure 2) did not show significant differences among the different groups tested (P>0.05).

## 4. Discussion

The main objective of the study was to evaluate the ability to control the buccolingual inclination of upper incisors with three different orthodontic appliances: conventional brackets, self-ligating braces, and aligners. The null hypothesis of the present report was accepted.

Variations of 11^∧^SnaSnp and 11^∧^Ocl angles have been evaluated. These angles measure the inclination of the upper incisors, respectively, on the bispinal plane and on the occlusal plane. The values measuring their modification over treatment were compared. For both angles, conventional brackets showed the highest numeric variation [6.11 and 6.88 degrees, respectively]. Lower values were reported with self-ligating devices [5.64 and 5.12 degrees, respectively]. The lowest data were reported with aligners [5.13 and 4.60 degrees, respectively]. However, the differences among various techniques were not significant for both angles. Moreover, also the variation during treatment of the linear measurement I+ TVL showed no significant difference among the three different techniques. In the present study, all the data were measured with cephalometric software that allows an increased precision in landmarks localization and provides decimal rounded measurements, easing an accurate evaluation and comparison of clinical characteristics [19, 20]. In our knowledge, this is the first clinical study evaluating the buccolingual inclination of upper incisors of patients treated with different modalities [conventional brackets, self-ligating appliances, and aligners].

The angular [11^∧^SnaSnp and 11^∧^Ocl] and linear [I+ TVL] modifications of the radiographic position of the upper incisor occurring during treatment are important indicators to measure the clinical reliability and efficiency of orthodontic appliances in controlling upper incisors. Poor variations of these indicators may result from information loss due to devices inaccuracy [1].

The first orthodontic technique studied in the present report was the conventional multibrackets appliance. In this system, torque control depends on many clinical factors such as initial tooth inclination, vertical bracket position, and tooth anatomy [5]. Orthodontic bracket manufacturers have tried to improve the control of these factors through an elongation of bracket slot relative to the bracket base. Moreover, many other factors are involved: bracket design, mode of ligation, bracket deformation, wire stiffness, bracket width, and finally wire/slot play [engagement angle]. In fact, the 3D position of dental elements occurs as a result of the interaction between preformed arches and brackets on teeth with periodontal integrity support [21, 22]. The full expression of the torque can be potentially obtained using archwire with appropriate size that fill the bracket slot to achieve a close contact between wire and slot [23]. To insert a full-size rectangular arch, there is a need for a certain amount of play, and this means that the height of the bracket slot must be greater than the arch height. However, if the discrepancy between these two dimensions is excessive, there are inconsistencies in the output torque [21]. The loss of torque between an arch of 0.019x0.025” section [usual size for the final stages of orthodontic treatment] and a 0.022x0.028” slot is about 10°. Sometimes this difference between wire and slot size can be even bigger, because measures declared by the manufacturers of these appliances do not match the real ones [22, 24]. In this clinical study, torque expression for the orthodontic multibrackets appliances has been evaluated using a 0.019x0.025” stainless steel arch. It may be interesting in the future to test torque control with higher section wires [e.g., 0.021x0.025”]: it could enable analyzing the reduction effect of the bracket play in vivo [25].

The second orthodontic devices evaluated in the present report were self-ligating brackets. These stainless steel brackets are characterized by the presence of a fourth moving wall that converts the slot into a tube [11]. Self-ligating brackets are primarily designed to reduce the friction forces generated between wire and ligature during dental shifting [12]. In a fixed orthodontic treatment, 50% of the forces applied are used to overcome the friction [26]. These devices are claimed to reduce also treatment time and patient pain, but these concerns are still controversial [27]. The basic advantage of these brackets involves the elimination of elastomeric or metallic modules of ligation, along with the process or tools associated with their application. Therefore, the most important effect in treatment is supposed to be the achievement of consistent wire engagement without the undesirable force relaxation of elastomeric modules. This feature should guarantee a constantly active status of engaged wires [28]. Some authors analyzed the incisor torque of self-ligating brackets in vitro tests. The results showed that conventional brackets presented better torque control than self-ligating, even with the same arch and slot dimensions [4, 29]. These general outcomes also apply to the present study: in fact, incisal torque expression of self-ligating devices has been demonstrated to be lower than conventional brackets. The 11^∧^SNASNP and 11^∧^Ocl variations were lower than conventional brackets, even if the differences were not statistically significant.

The third technology studied in this research was aligners. This technique has some considerable clinical advantages. In fact, these devices allow satisfying the patient's aesthetic demand, due to their small size and transparency [30]. Moreover, oral hygiene procedures are simpler and faster with no fixed bracket bonded to teeth surface [16, 28]. However, torque expression is particularly complex to control using a removable device [17]. To overcome this characteristic, the Power Ridge feature was introduced as an appropriate altered geometry of the conventional aligners. This system is a twist of the aligner surface designed to maintain a correct fit at gingival margin during tooth movement. This condition allows controlling the couple of forces and helps tooth movement around its center of resistance. It is usually built into the case set-up when at least 3 degrees of lingual root torque are required [31].

The measurements of the buccolingual inclination in the digital world allowed previous authors to state that, when a mean root torque information of 10.4° was required for an upper central incisor, an expression of the 99% of the third-order information was detected, demonstrating a negligible torque loss [31]. Despite the widespread of the technique, no other studies are available. However, several authors analyzed the ability of aligners in controlling the buccolingual inclination of several teeth comparing aligners and conventional fixed appliances. In a study based on cone beam computed tomograms [CBCT], Grunheid et al. showed a significantly high value of buccolingual inclination for mandibular canines with aligners compared with fixed appliance treatment [2.6° of difference] [32].

Other authors analyzed cephalometric position of mandibular incisors [33]. In mild-to-moderate anterior crowding cases, there were no changes in the position or angulation of the mandibular incisors. In severe anterior crowding, mandibular incisors showed a higher buccal inclination [L1-NB: -4,7°; L1-NB: -1.55 mm; L1-APog: -4,82°; L1-APog: -1.74 mm]. In another RCT, no differences in mandibular incisors buccal inclination produced by aligners or fixed labial appliances treatment in mild crowding cases were shown [34].

When evaluating torque expression of orthodontic appliances also the age of the patients has to be carefully considered. In fact it is well known that torque and overjet change remarkably during certain growth phases [35, 36]. In the present report mean age of the patients was 25 years old, in order to avoid torque variability due to subject growth.

Finally, the existing literature is mainly focused on materials and methodological aspects of aligner orthodontics, while only in the last years the interest in evaluating the efficiency of orthodontic movements obtained by these devices has grown very fast. However, the number of studies comparing the effects of different orthodontic techniques, including the aligners, is very low. Other studies have evaluated torque control efficiency of different orthodontic technique, analyzing the position of teeth in vitro [8, 10, 25]. To our knowledge, unfortunately no clinical research has been carried out by mean of cephalometric assessment; therefore a direct comparison with the results of this present investigation is not feasible.

This study showed that both aligners and self-ligating brackets generated lower torque control if compared to conventional brackets, but these differences were not statistically significant different. Further studies are needed to confirm or disprove the results of this retrospective research.

## 5. Conclusions

When evaluating radiographic angular measurements during treatment, 11^∧^SnaSnp and 11^∧^Ocl angles showed the highest numeric variation with conventional brackets. Lowest values were reported with aligners. However, the differences among various techniques were not statistically significant for both angles. Also I+ TVL linear value variation did not show significant differences among the different groups tested.

Based on these results, all the three different systems showed good clinical reliability in the upper incisor torque control.

## Figures and Tables

**Table 1:** Pretest patients homogeneity evaluation.

**Pretreatment values**	**Appliance**	**Mean**	**SD**	**Min**	**Median**	**Max**	**Significance ** *∗*
11∧SNASNP	Conventional	110.31	5.95	94.80	110.25	121.00	A
	Self-ligating	111.48	6.07	96.30	112.60	124.70	A
	Aligner	109.53	6.47	94.10	108.70	123.00	A

11∧Ocl	Conventional	62.00	7.65	47.10	58.50	79.20	B
	Self-ligating	60.13	6.94	45.50	61.00	75.40	B
	Aligner	63.70	6.65	48.30	62.40	78.20	B

I+ TVL	Conventional	2.27	1.57	0.20	1.90	5.60	C
	Self-ligating	2.31	1.87	0.10	2.00	6.80	C
	Aligner	2.33	1.09	0.00	1.90	5.20	C

ANB	Conventional	4.18	1.58	0.50	4.10	7.30	D
	Self-ligating	4.05	1.40	0.60	4.30	6.10	D
	Aligner	4.16	2.04	0.40	4.45	8.20	D

WITS	Conventional	2.25	1.61	0.00	1.70	6.90	C
	Self-ligating	2.30	1.42	0.00	2.00	6.40	C
	Aligner	2.43	1.79	0.50	1.90	7.70	C

SNASNP^∧^GOME^ ^^ ^	Conventional	25.74	5.49	15.30	26.05	35.50	E
	Self-ligating	25.40	4.05	13.90	24.20	31.90	E
	Aligner	26.96	4.70	15.80	26.85	35.80	E

*∗*: Tukey significance. Means with the same letters are not significantly different.

**Table 2:** Evaluation of variation in incisal inclination using the three different orthodontic techniques.

Variable	Appliance	Mean	SD	Min	Median	Max	Significance *∗*
11^∧^SNASNP	Conventional	6.11	3.91	0.10	5.75	14.20	A
	Self-ligating	5.64	3.27	0.50	5.70	18.70	A
	Aligner	5.13	3.23	0.40	3.75	15.40	A

11^∧^OCL	Conventional	6.88	4.28	0.10	6.95	20.90	B
	Self-ligating	5.17	3.10	0.00	3.50	15.50	B
	Aligner	4.60	3.46	0.20	3.85	15.40	B

I+ TVL	Conventional	1.56	0.47	0.00	1.10	4.60	C
	Self-ligating	1.62	0.66	0.10	1.70	6.20	C
	Aligner	1.47	0.57	0.20	1.25	6.10	C

*∗*: Tukey significance. Means with the same letters are not significantly different.

## Data Availability

The data used to support the findings of this study are available from the corresponding author upon request.
